# Source attribution of salmonellosis by time and geography in New South Wales, Australia

**DOI:** 10.1186/s12879-021-06950-7

**Published:** 2022-01-04

**Authors:** Angus McLure, Craig Shadbolt, Patricia M. Desmarchelier, Martyn D. Kirk, Kathryn Glass

**Affiliations:** 1 National Centre for Epidemiology and Population Health, Australian National University, Canberra, Australia; 2 New South Wales Department of Primary Industries, New South Wales, Australia; 3 Food Safety Principles, Brisbane, Australia

**Keywords:** *Salmonella* infections, Source attribution, Foodborne disease, Gastroenteritis, Disease reservoir, Zoonosis, Bayesian analysis

## Abstract

**Background:**

*Salmonella* is a major cause of zoonotic illness around the world, arising from direct or indirect contact with a range of animal reservoirs. In the Australian state of New South Wales (NSW), salmonellosis is believed to be primarily foodborne, but the relative contribution of animal reservoirs is unknown.

**Methods:**

The analysis included 4543 serotyped isolates from animal reservoirs and 30,073 serotyped isolates from domestically acquired human cases in NSW between January 2008 and August 2019. We used a Bayesian source attribution methodology to estimate the proportion of foodborne *Salmonella* infections attributable to broiler chickens, layer chickens, ruminants, pigs, and an unknown or unsampled source. Additional analyses included covariates for four time periods and five levels of rurality.

**Results:**

A single serotype, *S.* Typhimurium, accounted for 65–75% of included cases during 2008–2014 but < 50% during 2017–2019. Attribution to layer chickens was highest during 2008–2010 (48.7%, 95% CrI 24.2–70.3%) but halved by 2017–2019 (23.1%, 95% CrI 5.7–38.9%) and was lower in the rural and remote populations than in the majority urban population. The proportion of cases attributed to the unsampled source was 11.3% (95% CrI 1.2%–22.1%) overall, but higher in rural and remote populations. The proportion of cases attributed to pork increased from approximately 20% in 2009–2016 to approximately 40% in 2017–2019, coinciding with a rise in cases due to *Salmonella* ser. 4,5,12:i:-.

**Conclusion:**

Layer chickens were likely the primary reservoir of domestically acquired *Salmonella* infections in NSW circa 2010, but attribution to the source declined contemporaneously with increased vaccination of layer flocks and tighter food safety regulations for the handling of eggs.

**Supplementary Information:**

The online version contains supplementary material available at 10.1186/s12879-021-06950-7.

## Background

Salmonellosis is a common cause of foodborne illness and hospitalisation across Australia. Annual notification rates for salmonellosis in Australia’s most populous state, New South Wales (NSW), ranged between 38.1 and 57.9 per 100,000 population during 2009–2019 [1]. However, for every notified case of salmonellosis, there are an estimated seven cases that go unreported [2]. In 2015, there were an estimated 64 thousand annual episodes of foodborne salmonellosis in Australia that resulted in three thousand hospitalisations and thirteen deaths. The estimated annual societal cost of this illness was AUD 105 million [3–5].

*Salmonella* can be found in all major livestock and poultry species and a wide array of wild animals; however, the proportion of human *Salmonella* infections attributable to each of these sources is population- and geography-dependant and often unknown. For instance, in the Netherlands, the primary sources were layers and pigs [6], in Italy and Denmark the primary sources were pigs [7, 8], while in Sweden over 80% of cases were attributed to travel [9]. Source attribution analyses in the Australian states of South Australia and Queensland both identified chickens (broilers and layers) to be the primary sources of illness [10, 11], but also highlighted attribution to nuts or environmental exposures in sub-tropical Queensland [10, 12]. Estimates of the proportion of infections arising from each source can highlight areas needing intervention and inform biosecurity and food safety policy across the entire food supply chain. For instance, a source attribution study in New Zealand in the 2005 demonstrated that > 50% of *Campylobacter* infections were attributable to poultry, prompting targeted improvements to the food safety standards and practices around poultry. Following these interventions, annual campylobacteriosis notification rates in 2008 declined by 54% compared to 2000–2006 [13] and remained stable over the following decade [14]. Further source attribution analyses demonstrated the decline was due primarily to a 74% reduction in cases attributed to poultry [13]. In NSW, a broad range of interventions were put in place to prevent foodborne salmonellosis following the release of the new NSW Food Safety Strategy 2015–2021. Though salmonellosis notification have declined from approximately 58 per 100,000 in 2014 to 42 per 100,000 in 2018 [1], the proportion of foodborne salmonellosis attributable to different animal reservoirs before and after this period was unknown.

*Salmonella enterica* subsp*. enterica,* which accounts for nearly all human cases of salmonellosis, can be classified into over 2500 serotypes [15]. Some serotypes are adapted to specific host species, such as *Salmonella* ser. Typhi, which has humans as the primary host and *Salmonella* ser. Gallinarum, which has poultry as the primary host [16]. Other serotypes can be found in one or more animal hosts and can cause human infections e.g. *Salmonella* ser. Typhimurium. *Salmonella* can be further differentiated using a range of typing schemes, including phage typing, multi-locus sequence typing (MLST), multiple-locus variable-number tandem repeat analysis (MLVA), whole genome sequencing, and other techniques [15]. Although it has become more common to type *Salmonella* using MLVA or whole genome sequencing for their superior ability to differentiate types, serotyping remains a standard typing method for *Salmonella* isolates from animals and humans in Australia.

Several methods have been developed to attribute illness to specific sources [17, 18]. Some rely on linking individual cases or outbreak cases to putative sources through epidemiological investigations, including case–control studies. However, these approaches are labour-intensive and often inconclusive due to small sample sizes. A suitable approach for sporadic cases that does not require food questionnaires is to compare the distribution of *Salmonella* types in isolates from cases to the distribution of types in putative sources. This approach estimates the overall proportion of cases attributable to each source. Hald et al. adopted a Bayesian source attribution framework [7] and their model and its variants have since been applied widely [10–12, 19–21]. A related approach accounts for genetic relatedness of strains using the asymmetric island model [21, 22]. Recent advances allow these models to be adjusted for covariates to identify trends or differences by subgroup [21]. For instance, recent studies of campylobacteriosis in New Zealand have demonstrated that attribution proportions to different sources vary between rural and urban areas [21]. In Australia, the spatial distribution of salmonellosis varies by serotype (e.g. *Salmonella* ser. Wangata is associated with proximity to wetlands [23]), suggesting that the proportion of cases attributable to different animal sources may also vary spatially.

We conducted a source attribution analysis for *Salmonella* infections acquired in New South Wales, Australia between January 2008 and August 2019 using variants of the Hald approach. We adjusted these models to account for trends over time and considered differences by age-group, gender, and rurality.

## Methods

### Human data

In NSW, treating pathology laboratories are required under public health legislation to report all *Salmonella* infections to the health department. We used deidentified data for 40,837 human cases notified between January 2008 and August 2019. The available case data included: typing information for the *Salmonella* isolates, patient gender, 5-year age group, location, and travel history. While nearly all isolates were characterised by serotype, only a minority of these isolates were further characterised with MLVA or phage typing. Location was encoded at the Statistical Area 2 (SA2) level as defined in the Australian Statistical Geography Standard. As we were primarily interested in cases acquired in NSW, we removed all cases recorded as being acquired outside NSW. However, travel history was known only for a small minority of cases, and before May 2010, cases that were known not to have travelled and cases with unknown travel history were recorded identically. Therefore, we also excluded all cases due to serotypes deemed to be *travel-associated*. A serotype was deemed to be travel-associated if more than half of cases after May 2010 with a known travel history were believed to be acquired outside NSW.

### Non-human data

Data for non-human sources were collated from the National Enteric Pathogens Surveillance System (NEPSS) and the New South Wales Food Authority (NSWFA). The two datasets included non-human *Salmonella* isolates sampled from animals, animal products, farm environments (e.g. animal pens and barns), food, and the natural environment. Isolates considered as from potential animal sources were those derived from the faeces, carcass, or enclosures of an animal or from an animal product. As with human isolates, nearly all isolates were characterised by serotype, but only a minority of these isolates were further characterised with MLVA or phage typing. Isolates that were not serotyped or were from a travel-associated serotype were excluded. We also excluded isolates without a recorded date or with a date before 2008.

Non-human animal isolates were categorised by reservoir animal source where possible from the recorded details. For example, all isolates from pig carcasses; pig faeces; cooked, cured, or raw pig meats; pig offal; pig intestinal contents; and pig farm environments (e.g. bedding) were categorised as *porcine*. Broiler and layer chickens were treated as separate reservoirs. Chicken meat products for human consumption and environmental samples from broiler farm premises and chicken meat processors were categorised as *broiler*, while isolates from eggs, egg products (e.g. mayonnaise), and egg farm premises were categorised as *layer*. Isolates that could not be definitively assigned to an animal reservoir were excluded, e.g. feather meal which might have been derived from broilers or layers and fresh produce which might have been contaminated directly or indirectly by any wild or domestic animal. We excluded isolates from food products with multiple animal origins (e.g. ‘egg and bacon roll’) and isolates with ambiguous descriptions (e.g. ‘meat’). Where possible, putative sources were combined to form categories with at least 100 isolates. Putative sources that could not be reasonably combined into broader categories with at least 100 isolates were excluded.

As source attribution analysis requires the same typing scheme to applied to both source and human data and serotyping was the most consistently and completely applied method, analyses were conducted using serotyped data and isolates without serotyping information were removed from both the human and non-human datasets.

### Source attribution modelling framework

We generalised existing Bayesian source attribution methods [20, 21] to estimate changing attribution proportions over time, include covariates for the cases, and adjust for differences between types.

The proportion (${\theta }_{ijst}$) of cases in subpopulation $s$ during period $t$ that were due to type $i$ from source $j$ was modelled as:

$$\theta_{ijst} \propto a_{jst} w_{j} r_{ijt} q_{i}$$

with constraints ${\sum }_{i,j}{\theta }_{ijst}=1$ and ${\sum }_{i}{r}_{ijt}=1$ and where ${a}_{jst}$ was the ability of source $j$ during period $t$ to act as a reservoir of infection for group $s$, ${w}_{j}$ was a weight for the relative exposure of humans to contamination from source $j$, ${r}_{ijt}$ was the relative prevalence of type $i$ in source $j$ during period $t$, and ${q}_{i}$ was the relative ability of subtype $i$ to lead to human infection. In each group $s$ and period $t$ the proportion of infections of all types attributed to a source, ${\xi }_{jst}$, was:

$$\xi_{jst} = \mathop \sum \limits_{i} \theta_{ijst} \propto a_{jst} w_{j} \mathop \sum \limits_{i} r_{ijt} q_{i} ,$$

while the proportion of cases due to each type, ${\mu }_{ist}$, was:

$$\mu_{ist} = \mathop \sum \limits_{j} \theta_{ijst} \propto q_{i} \mathop \sum \limits_{j} a_{jst} w_{j} r_{ijt} .$$

The estimation of these parameters occurred in two steps. The distribution of types in each source and period (${r}_{ijt}$) were estimated first, and all other parameters were then estimated repeatedly with draws from the posterior distribution of ${r}_{ijt}.$ In the first step, the number of isolates of each type observed in each source (${X}_{ijt}$) were modelled with multinomial distributions in one of two ways. For sources with many isolates in every period, the relative frequency of types were modelled independently for each period based only on the data collected in that period, i.e. ${X}_{jt} \sim Multinomial\left({r}_{jt}\right)$. With a unit Dirichlet prior this resulted in a Dirichlet posterior distribution: $p\left({r}_{jt}|X\right) \sim Dirichlet\left(1+{X}_{jt}\right).$ For sources with too few samples in each period, the data across the whole study was used for every time period resulting in posterior estimates: $p\left({r}_{jt}|X\right) \sim Dirichlet\left(1+{\sum }_{\tau }{X}_{j\tau }\right).$

In estimating the remaining parameters, the efficiency of each type (${q}_{i}$) and the exposure weights (${w}_{j}$) were assumed to remain constant over time but source efficiencies (${a}_{jst}$) were allowed to vary over time and by subgroup of cases. This was modelled as:

$$a_{jst} = \exp \left( {\tau_{tj } + \mathop \sum \limits_{n} F_{sn} \beta_{nj} } \right).$$

where $F$ was a matrix defining a linear predictor based on categorical, ordinal, or continuous covariates for each subgroup (i.e. category of a covariate) $s$ of the cases; $\beta$ was a matrix of parameters for each source $j$; and $\tau$ defined temporal differences in source efficiency by time $t$ and source $j$. A reference group was assigned to each covariate, and the associated parameters fixed to 0, while the remaining parameters are given unit normal priors.

The number of human cases in subpopulation $s$ during period $t$ that were due to pathogen type $i$, were modelled as independent multinomial variables for each period $t$ and subgroup $s$, i.e. ${Y}_{st} \sim Multinomial\left({\mu }_{st}\right)$. The ${q}_{i}$ were constrained with a hierarchical log-normal prior:

$$\begin{aligned} p\left( {q_{i} |\sigma } \right) & \sim  lognormal\left( {0,\sigma^{2} } \right) \\ p\left( \sigma \right) & \sim Uniform\left( {0, 5} \right). \\ \end{aligned}$$

For predominantly foodborne infections the exposure weights ${w}_{j}$, can be approximated by the relative level of exposure to contaminated food products derived from each source. However, as this is not measured directly, we modelled this as ${w}_{j}={M}_{j} {k}_{j}$, where ${M}_{j}$ was the per capita consumption of food derived from source $j$, and ${k}_{j}$ was the prevalence of the pathogen in food derived from source $j$, which was estimated from surveys of animal food products. For each source $j$, we modelled the number of total tests (${N}_{j}$) and positive tests (${P}_{j}$) ${P}_{j} \sim Binomial\left({N}_{j}, {k}_{j}\right),$ with an uninformative uniform prior on prevalence, i.e. ${p(k}_{j}) \sim Beta\left(\mathrm{1,1}\right).$

Our model framework was extended to include an ‘unsampled source’ by including an additional source ${j}^{*}$ with no observed samples, i.e. ${X}_{i{j}^{*}t}={P}_{{j}^{*}}={N}_{{j}^{*}}=0$.

### Source attribution models

As a base case we considered a model with no covariates and no temporal variation, which was equivalent to the Modified Hald model applied to the whole study period [20]. As a sensitivity analysis we compared this to a model where all the type efficiency terms ${q}_{i}$ were fixed at one, which was equivalent to the Dirichlet model of Liao et al. [21] with the addition of exposure weights. We considered each model with and without an ‘unsampled source'.

We then considered models with combinations of covariates (age, rurality, and gender) with and without temporal variation. In models adjusting for age, we used age categories: 0–4, 5–19, 20–34, 35–64, and 65 and over. The rurality of each case was determined by matching the Australian Bureau of Statistics 2011 Statistical Area 2 (SA2) code provided for the case to one of the five rurality zones (Major Cities, Inner Regional, Outer Regional, Remote, and Very Remote) defined in the 2011 Australian Statistical Geography Remoteness Structure. However, as rurality was defined at the more granular SA1 level and each SA2 is built from SA1s, a few SA2s contained regions with different categories of rurality. Cases associated with these SA2s were assigned the rurality zone closest to the average rurality of the constituent SA1s. In our main analyses, rurality was modelled as an ordinal variable. In a sensitivity analysis we modelled rurality as a nominal categorical variable, combining the five categories down to three categories (‘Major Cities’, ‘Regional’ and ‘Remote’) to increase the number of cases in each category. The small number of cases that had a missing value for gender, age, or location were excluded only from analyses involving the missing covariate.

In models with temporal variation, we considered four three-year periods: 2008–2010, 2011–2013, 2014–2016, and 2017–2019. As there were > 200 isolates per period from broilers and > 450 isolates per period from layers, the relative frequency of serotypes was estimated independently for each period for broilers and layers. As there were relatively few isolates for ruminants and pigs in at least one period (< 30 for pigs and < 20 for ruminants), data across all study years was used to estimate the relative frequency of serotypes in ruminants and pigs in every period.

To improve model convergence, we excluded serotypes that rarely caused disease in humans (i.e. < 10 human cases across the study period) from the analysis.

### Exposure by source

Table 1 provides a summary of prevalence assumptions with references, while Table 2 summarises assumptions on food consumption per capita. The datasets used to determine the number of samples positive for each pathogen type ${(X}_{ijt})$ did not include data on the number of total tests, so the prevalence of *Salmonella* in food products was estimated from separate surveys, with an adjustment to reduce the number of positive samples by the fraction of cases from excluded serotypes from each source. As age, sex, location, and time-specific consumption and prevalence data were not available for all food sources, exposure weights and prevalence on animal products were assumed fixed across all time periods and the whole population. We used published national data on *Salmonella* from the *E. coli* and *Salmonella* monitoring (ESAM) program from the early 2000s to inform prevalence in ruminants and pork [24, 25], as publicly available contemporary surveys in NSW had insufficient samples to determine prevalence. The relative exposure of the human population to ruminants, pigs, and broilers were based on the apparent consumption of meats from these sources published by the Australian Bureau of Agricultural and Resource Economics and Sciences (ABARES) [26]. For eggs, we assumed consumption of one egg was equivalent to 200 g of meat (as in our previous work [11]) and used consumption estimates from a 2018 Australian Eggs annual report [27]. In models with an ‘unsampled’ source we adopted the conservative assumption that exposure to the unsampled source was equal to the source or group of sources with the highest consumption.

**Table 1:** *Salmonella* prevalence assumptions by source with references

	Prevalence (95% CI)	Prevalence adjusted for types rare in cases (95% CI)	References
Chicken meat	48.4% (42.0–54.8)	38.6% (32.5–45.0)	NSW-specific data in Table 4 [50]
Chicken eggs	1.76% (0.70–3.59)	1.44% (0.66–2.72)	Following prior assumptions [11]
Pigs	1.88% (1.57–2.22)	1.14% (0.90–1.41)	National ESAM^a^ data [24]
Ruminants	0.38% (0.33–0.43)	0.37% (0.32–0.42)	National ESAM^a^ data [25]

^a^*E. coli* and *Salmonella* monitoring program

**Table 2:** Relative exposure to potential sources of *Salmonella*, measured by mean consumption of meat and animal products per person per year in Australia

	Relative Exposure (kg/person/year or equivalent)	References
Chicken meat	47	ABARES^a^ [26]
Chicken eggs	49^b^	Australian eggs [27]
Pork	28	ABARES^a^ [26]
Ruminants	34	ABARES^a^ [26]

^a^Australian Bureau of Agricultural and Resource Economics and Sciences

^b^Mean consumption was approximately 245 eggs per person per year in the 2017–2018 financial year. The relative exposure was calculated assuming that one egg is equivalent to 200 g of meat

### Software

All analyses were conducted in the R programming environment [28]. Bayesian inference was performed using the No U-turn Algorithm with the Stan programming language [29] and the rstan R package [30]. Data cleaning and manipulation was done using the plyr [31], dplyr [32], and tidyr [33] R packages. Data visualisations were made with the ggplot2 R package [34].

### Ethical approval and consent to participate

All methods were carried out in accordance with a protocol approved by the Australian National University (ANU) Human Research Ethics Committee (Protocol: 2019/470) and in accordance with all ANU guidelines. The data on human infections were collected under the NSW Public Health Act 2010 and provided to us in a de-identified format by NSW Health. Under the Act, medical practitioners and laboratories were required to notify or report cases of Salmonella infections to NSW public health units.

## Results

### Data

After excluding isolates from humans with no serotype information (3807 isolates, 9.3%), isolates from cases recorded as having travelled outside NSW (2381 isolates, 5.8%), isolates of *Salmonella* ser. Paratyphi B bv Java and from serotypes that were commonly travel associated (4089 isolates, 10.0%), and isolates of serotypes that were rare in humans (487 isolates, 1.2%), a total of 30,073 human isolates remained for source attribution analysis (Table 3). Most of the cases associated with these isolates were reported in major cities, with 51% of cases in females. After isolates from some animals were grouped and others were omitted (e.g. wildlife, horses, ducks, quail, and turkeys), the final source groups were broilers (N = 1396), layers (N = 2321), pigs (391), and ruminants (cattle, sheep, and goats; N = 435) (Table 4). The number of non-human isolates in our dataset was lowest in 2017–2019.

**Table 3:** Characteristics of *Salmonella* cases reported between January 2008 and August 2019 in New South Wales after excluding cases without known serotype (N = 3807) travel associated cases (N = 6470) and rare serotypes (N = 487)

	*S.* Typhimurium (N = 18,802)	Other serotypes (N = 11,271)	All serotypes (N = 30,073)	p value
Gender				0.010
Missing	47	22	69	
Female	9680 (51.6%)	5633 (50.1%)	15,313 (51.0%)	
Male	9075 (48.4%)	5616 (49.9%)	14,691 (49.0%)	
Rurality				< 0.001
Missing	142	48	190	
Major cities	14,106 (75.6%)	7521 (67.0%)	21,627 (72.4%)	
Inner regional	3434 (18.4%)	2784 (24.8%)	6218 (20.8%)	
Outer regional	1070 (5.7%)	844 (7.5%)	1914 (6.4%)	
Remote	30 (0.2%)	48 (0.4%)	78 (0.3%)	
Very remote	20 (0.1%)	26 (0.2%)	46 (0.2%)	
Age group				< 0.001
Missing	17	6	23	
00–04	4191 (22.3%)	3045 (27.0%)	7236 (24.1%)	
05–19	4186 (22.3%)	1563 (13.9%)	5749 (19.1%)	
20–39	4986 (26.5%)	2287 (20.3%)	7273 (24.2%)	
40–64	3434 (18.3%)	2445 (21.7%)	5879 (19.6%)	
65 +	1988 (10.6%)	1925 (17.1%)	3913 (13.0%)	
Year group				< 0.001
2008–2010	4780 (25.4%)	2059 (18.3%)	6839 (22.7%)	
2011–2013	5441 (28.9%)	2407 (21.4%)	7848 (26.1%)	
2014–2016	5861 (31.2%)	3457 (30.7%)	9318 (31.0%)	
2017–2019	2720 (14.5%)	3348 (29.7%)	6068 (20.2%)

**Table 4:** The number of serotyped *Salmonella* isolates from humans and selected non-human sources sampled in NSW between January 2008 and June 2019

Source	Year												Total
	2008	2009	2010	2011	2012	2013	2014	2015	2016	2017	2018	2019	
Human cases	1759	2101	2979	2864	2275	2709	3416	2818	3084	2489	2063	1516	30,073
Ruminants	58	60	49	87	60	33	25	42	10	6	2	3	435
Cattle	50	53	40	70	53	31	22	38	9	6	2	3	377
Ruminants—Other^a^	8	7	9	17	7	2	3	4	1	–	–	–	58
Broilers^b^	152	99	98	76	117	37	61	360	185	84	56	71	1396
Layers^b^	288	160	155	270	204	251	225	158	109	136	128	237	2321
Pigs	20	13	42	47	45	79	23	48	51	5	14	4	391
Total Included Non-Human^c^	518	332	344	480	426	400	334	608	355	231	200	315	4543
Wildlife^d^	12	16	1	3	22	13	5	5	3	3	–	–	83
Poultry—Other^e^	13	7	1	9	1	4	11	1	1	–	–	–	48
*Horses*	2	3	4	7	1	0	4	8	3	–	–	–	32

These counts exclude *S*. Paratyphi B bv Java, types that were rare in humans (less than 10 cases over the period), and travel-associated types (types where more than half of cases with known travel history were acquired outside NSW). A selection of the most common excluded sources are displayed here to illustrate the sparsity of data over time

^a^* Ruminant-other* includes sheep and goats

^b^Other isolates from poultry that could not be linked to either broilers or layers are not included in this table

^c^The included sources are *ruminants, broilers, layers,* and *pigs*

^d^*Wildlife* consists of a diverse group of non-captive non-domesticated animalsTyphimurium

^e^*Poultry-other* consists of ducks, turkey and quail

### Type distribution in sources and humans

*S.* Typhimurium was the most common serotype in cases and every major source group except for broilers (Additional file 1: Fig. S1, Table S1). In broilers, *S.* Typhimurium was only the third most common serotype (10.1%, 141/1396) after *Salmonella* ser. Infantis (30.4%, 425/1396) and *Salmonella* ser. Sofia (20.6%, 287/1396) (Additional file 1: Fig. S1). The proportion of human cases due to *S.* Typhimurium was largest in major cities (65.2%), but this proportion declined with increasing rurality (Inner Regional 55.2%; Outer Regional 55.9%; Remote 38.5%; Very Remote 43%) (Table 3). Conversely, cases due to serotypes such as *Salmonella* ser. Chester and *Salmonella* ser. Saintpaul were less common in major cities than in regional and remote NSW. The proportion of human cases due to *S.* Typhimurium declined from 65 to 75% during 2008–2013 to < 50% during 2017–2019. The proportion of cases due to some serotypes rose over the same period, e.g. *S.* Wangata (2008–2010: 1.0%; 2017–2019: 9.2%)*,* and *S. enterica* ser. 4,5,12:i:- (2008–2010: 0.0%; 2017–2019: 5.5%). The proportion of layer isolates that were serotyped as *S.* Typhimurium also declined over the same period (2008–2010: 41.6%, 251/603; 2017–2019: 17.4%, 87/501).

### Subtyping

After restricting the analysis to serotyped isolates, only a small minority of non-*S.* Typhimurium isolates were also typed using phage or MLVA typing (human: 12.6%; reservoirs: 4.5%). While a larger proportion of *S.* Typhimurium isolates were typed using a least one additional method (human: 95.4%; reservoirs: 73.1%), this additional typing method was not consistent over time or between cases and reservoirs. Nearly all *S.* Typhimurium isolates from humans were phage typed prior to 2010, but < 7% were phage typed after 2010 (Additional file 1: Table S1). Overall, fewer *S.* Typhimurium isolates from humans were phage typed (25.1%) than in the major source groups (58.2–84.4%). In contrast, while 93% of *S.* Typhimurium isolates from cases were MLVA-typed, less than 35% of *S.* Typhimurium isolates in each major source group were MLVA-typed and coverage varied by year and source. We were therefore unable to use phage typing or MLVA data in our source attribution analyses.

### Source attribution without covariates

Table 5 summarises the attribution proportion for models without covariates or temporal variation. All models indicated layers as the leading source of illness. If we assumed all serotypes to be equally efficient at causing disease in humans, very few human cases were attributed to either broilers or pigs: 0.02% and 1.8% respectively. When allowing for differences between serotypes, a greater proportion of cases were attributed to broilers and pigs: 13.1% and 17.8% respectively. Equal-$q$ and variable-$q$ models with adjustments for unsampled sources attributed 5.8% and 11.3% to the unsampled source respectively and attributed fewer cases to ruminants and pigs than the models without adjustment for an unsampled source. The HaldDP model of Miller et al. [19] failed to converge with our dataset and is not shown.

**Table 5:** The percentage of human isolates attributed to ruminants, broilers, layers, pigs, and other unsampled sources with 95% credible intervals for models without covariates or temporal variation

	Ruminants	Broilers	Layers	Pigs	Unsampled
Equal-$q$					
without unsampled source	41.3 (21.0–58.7)	0.02 (0.0–0.05)	56.9 (39.1–74.2)	1.8 (0.0–8.3)	–
with unsampled source	36.0 (16.9–55.5)	0.01 (0.0–0.03)	57.7 (39.7–74.0)	0.4 (0.0–3.1)	5.8 (0.0–10.7)
Variable-$q$					
without unsampled source	24.7 (0.0–55.8)	13.1 (0.0–44.9)	44.3 (9.8–76.7)	17.8 (0.0–39.1)	–
with unsampled source	10.5 (0.0–34.8)	18.2 (0.0–66.1)	48.4 (5.4–79.6)	12.1 (0.0–31.8)	11.3 (1.2–22.1)

In the equal-$q$ model all *Salmonella* serotypes are assumed to be equally efficient in their ability to cause infection in humans, while in the variable-$q$ model (equivalent to the modified Hald model [20]) serotypes are allowed to differ in their efficiency

### Source attribution with covariates

**Fig. 1:**
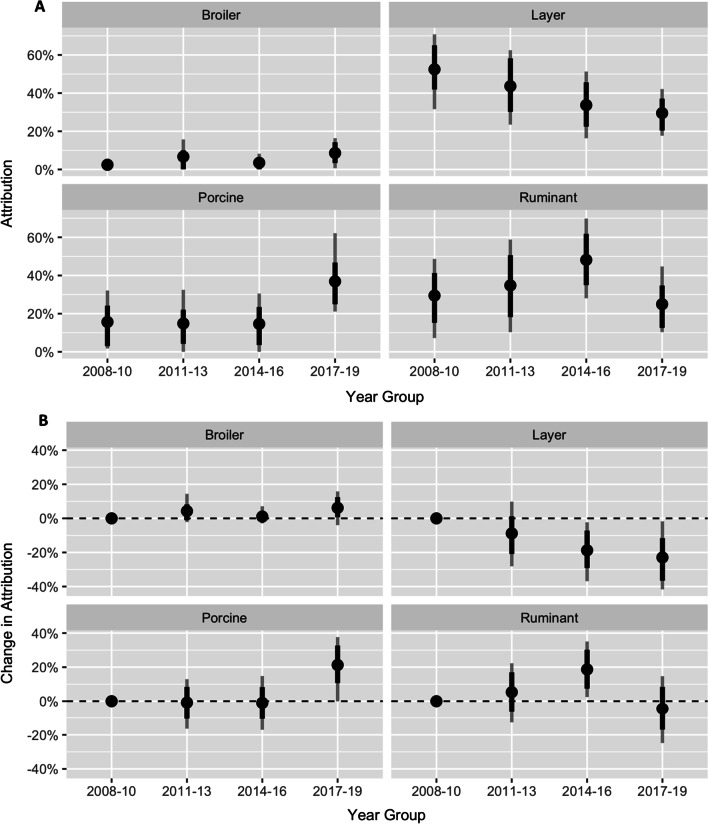
**A** Attribution proportion with 95% credible intervals for each of the major source groups for three-year periods and **B** change over time in the attribution proportion (in percentage points) with 2008–2010 as the reference. Dots indicate posterior mean, while dark and faint lines indicate 80% and 95% credible intervals respectively. The dashed horizontal line indicates no difference

**Fig. 2:**
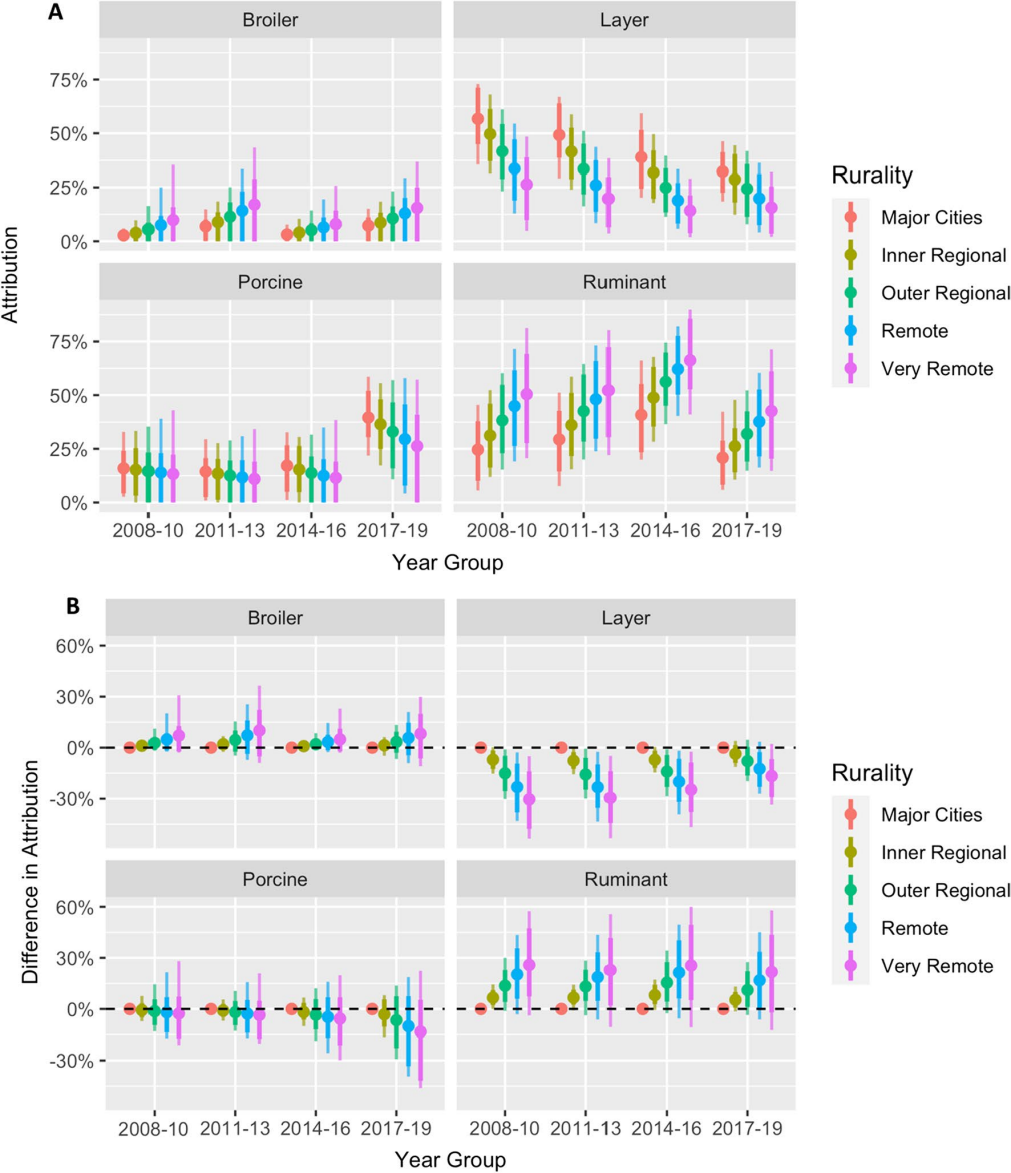
**A** Attribution proportion for each of the major source groups for cases residing in different rurality zones over time. **B** The difference in attribution proportion by rurality with residents of major cities as the reference. Dots indicate posterior mean, while dark and faint lines indicate 80% and 95% credible intervals respectively. The dashed horizontal line indicates no difference

In a temporal model without covariates, the proportion attributed to layers declined significantly from 55% (95% CrI 23–71%) in 2008–2010 to 34% (95% CrI 16–51%) in 2014–2016 and 30% (95% CrI 18–42%) in 2017–2019, with a significant increase in the proportion attributed to pigs in 2017–2019 and a significant increase in attribution to ruminants in 2014–2016 (Fig. 1). When rurality was included in the model, a significantly higher proportion of cases were attributed to layers in major cities, with this proportion declining with increasing rurality (Fig. 2). A similar rural–urban gradient was observed in a non-temporal model, although the proportion attributed to broiler chickens in that model (Additional file 1: Figure S2) was substantially higher than all other models, potentially due to confounding with year (p < 0.001 for association between year and rurality).

There was weaker evidence of a rural–urban gradient in attribution to ruminants, with a higher proportion of infections attributed to ruminants in rural areas (Figs. 2 and Additional file 1: S2), which disappeared in models with an unsampled source (Figures S3 and S4). In a sensitivity analysis where rurality was modelled as categorical variable with three levels (‘Major Cities’, ‘Regional’ and ‘Remote’) all findings remained qualitatively similar (results not shown).

A non-temporal univariable regression analysis found a significant association between attribution proportion and sex, with 5.7% (95% CrI 1.3%-10.1%) fewer male cases attributed to layer chickens. Though a qualitatively similar sex difference was also observed in a model including time and sex, and a model with age and sex, the sex differences were not statistically significant within any time period or age group. There were no consistent associations between age group and attribution proportions.

Estimates of the type-specific parameters, $q$, varied 1000-fold between the most efficient and least efficient serotypes. For instance, in the model including rurality, year-group, and an unsampled source, *S.* Typhimurium and *Salmonella* ser. Abortusovis were estimated to be 22.3 (95% CrI 14.0–38.0) and 0.19 (95% CrI 0.07–0.46) times as efficient as *S.* Infantis, while *S.* Sofia was estimated about as efficient as *S.* Infantis ($q$ ratio 0.5; 95% CrI: 0.2–1.7) (Additional file 1: Fig. S5).

## Discussion

We estimate that approximately half of all notified cases of non-typhoidal salmonellosis acquired in New South Wales during 2008–2019 were due to direct or indirect transmission from layer chickens. However, the proportion of cases attributed to layers declined from approximately 50% in 2009–2011 to approximately 25% in 2017–2019. The proportion of cases attributed to pork increased from approximately 20% in 2009–2016 to approximately 40% in 2017–2019, coinciding with a rise in cases due to *S. enterica* ser. 4,5,12:i:-, which has been found to be a persistent coloniser of pigs in Australia [35]. The proportion of cases attributed to layers was lower amongst rural-dwelling than urban-dwelling populations, similar to the rural–urban gradient found in a source attribution analysis of *Campylobacter* infections in New Zealand [21, 36].

The potential of zoonotic organism to lead to a notified case of foodborne disease depends on many factors including the organism’s ability to survive transport, storage, and food preparation and its virulence in humans. Some source attribution modelling approaches (e.g. those applied to *Campylobacter* infections [21, 22]) have assumed that all types are equally able to lead to notified human cases. However, this assumption is inappropriate for *Salmonella,* which is known to have both virulent (e.g. *S.* Typhimurium) and avirulent (e.g. *S.* Sofia [37]) serotypes. In our study, *S.* Typhimurium was more prevalent in cases (63%) than in broilers (10%), layers (32%), pigs (23%), or ruminants (35%), while *S.* Infantis was less prevalent in cases (3%) than in broilers (30%), layers, (19%) or pigs (7%), indicating that *S.* Typhimurium is a more efficient foodborne pathogen than *S.* Infantis. Consequently, models that assumed all serotypes were equally efficient attributed < 0.1% of cases to broilers (in which *S.* Sofia and *S.* Infantis were most common). However, this is inconsistent with the common implication of chicken meat in outbreak investigations and case control studies (e.g. [38]). Although models that account for differences between serotypes led to source attribution estimates with much wider confidence intervals, we believe they are more appropriate for modelling source attribution of *Salmonella*.

The decline in attribution to layers from 2014 onwards may be the result of interventions including vaccination of layers against *S.* Typhimurium. The NSW Food Safety Strategy 2015–2021, released in 2014, included a specified 30% reduction target for foodborne salmonellosis [39]. Prior to 2014, many reported foodborne outbreaks in NSW were linked to poor handling of eggs and hygiene at retail food service (e.g. [40, 41]). Several measures were implemented to address this, including mandatory education and food safety training on the risks of raw egg use, improved cleaning and sanitising for retail businesses, guidelines for the safe use of eggs which made it an offence in NSW to prepare raw egg foods without adequate processing, and training and guidance for local council Environmental Health Officers to focus on areas of highest food safety risk during inspections. Since 2014, large egg producers across NSW have begun largescale vaccination of layer flocks against *S.* Typhimurium; estimates based on sales of vaccines indicate approximately 75% of commercial layer flocks were vaccinated in 2017–2019 (Dr. Christopher Morrow, personal communication, April 2021). The introduction of all these measures coincided with a marked decline in *S.* Typhimurium and total *Salmonella* incidence in NSW [42].

The models that included an ‘unsampled’ source attempted to quantify the plausible proportion of cases that could be attributed to any animal reservoirs other than domestic chickens, ruminants, and pigs. Since this approach necessarily drew inferences without information about of the relative abundance of different serotypes in the ‘unsampled’ animal reservoirs, these models need to be interpreted cautiously. In our study, the increasing trend in attribution to ‘unsampled’ sources is associated with the increasing incidence of serotypes that were absent or rare in the major food animals included in our analyses, e.g. *S.* Wangata, *Salmonella* ser. Birkenhead, *Salmonella* ser. Waycross, and *Salmonella* ser. hvittingfoss. *S.* Wangata was not found in ruminants or pigs in our dataset and was very rare in broilers and layers (< 0.6%) but accounted for 9.0% of cases in 2017–2019 up from only 1.0% in 2008–2010. Similarly, though *S.* Birkenhead was only represented by a single isolate from layer chickens in 2019, the serotype accounted for 5.7% of cases in 2017–2019, up from 3.3% in 2008–2010. While *S.* Wangata is associated with contact with a range of wild animals in NSW [23, 43, 44], the animal reservoirs of *S.* Birkenhead, *S.* Waycross, and *S.* Hvittingfoss are unclear. A source attribution analysis of *Salmonella* infections in the neighbouring state of Queensland linked *S.* Birkenhead, *S.* Waycross, and *S.* Hvittingfoss to nuts (primarily locally grown macadamias) [10] suggesting environmental reservoirs of exposure. In north-eastern NSW, *S.* Birkenhead infection has been associated with not usually washing or peeling fruit and vegetables before eating raw. *S.* Hvittingfoss has caused outbreaks linked to contaminated cantaloupes in Australia with closely related isolates identified in bar-tailed godwits in north-western Australia, suggesting a common exposure to an unidentified animal or environmental reservoir [45]. *S.* Hvittingfoss has also been found in reptiles [46, 47], migratory ducks [48], and feral pigs [49] elsewhere in Australia. Consequently, a substantial part of the attribution to ‘unsampled’ sources in our model may be due to transmission from wild or feral animals through direct contact or indirect contamination of water, food, or the environment.

The present study has a number of strengths. The large dataset of *Salmonella* cases and a substantial dataset of isolates from major food animals, collected from across the state and over a long time period, have allowed us to identify the leading source of *Salmonella* infections, attribute separately to egg-laying and meat chickens, and identify temporal and rural–urban gradients in attribution. Furthermore, these key model outcomes were qualitatively robust across multiple model comparisons. In the source attribution framework we adopted attribution is to animal reservoirs rather than routes or vehicles of transmission. For instance, our estimate of the proportion of cases attributed to layers includes infections that may have been acquired through direct contact with layer chickens, consumption of contaminated eggs, contact with manure, or consumption of water or food contaminated by layer chicken faeces at any point between farm and table. Attribution to animal reservoirs is simultaneously a strength—since all possible transmission routes are captured—and a limitation—since the relative contribution of different routes cannot be quantified.

Our study has a number of limitations. No high-resolution molecular subtyping scheme was consistently applied to *Salmonella* isolates from humans and animals over the whole study period, forcing us to restrict our analyses to the level of serotype. Because the majority of human cases were due to a single serotype, *S.* Typhimurium, this restriction substantially weakened inferences, leading to wide confidence intervals for many attribution estimates. Though the dataset of reported human cases provided more than adequate sample size, it is estimated that as few as one in eight *Salmonella* cases are reported [2]. As unreported cases are likely to have milder symptoms than reported cases, more virulent serotypes of *Salmonella* are likely to be over-represented in our dataset and the attribution proportions are therefore likely indicative of moderate and severe cases. Similarly, rates of healthcare utilisation vary by rurality and other socioeconomic factors that may be associated with exposure to different animal sources, so attribution estimates may be biased towards those sources to which people with high healthcare utilisation rates are most exposed. Travel history was not available for all cases in our dataset, and though we took steps to remove cases not acquired in NSW from the study, we may have removed some locally acquired cases in doing so. The isolates from animal reservoirs were not collected in a single sampling frame. The majority of non-human *Salmonella* sampling by the NSW Food Authority occurred through investigation of foodborne disease outbreaks and was often sporadic in nature. Some targeted survey work was also undertaken, but this was biased towards testing of layer flocks, or opportunistic *Salmonella* testing through poultry meat verification work focused on *Campylobacter*. To date, NSWFA have not conducted comparable ongoing surveys or studies focused on ruminants, pigs, or other potential reservoirs. The remaining isolates from non-human sources collected by the NEPSS included any isolate submitted to *Salmonella* typing laboratories in Australia and included public, private, and academic sampling efforts. For isolates from non-human sources, we only considered those collected in NSW; however, NSW residents consume food produced across Australia. Though lamb and mutton account for approximately 8% of meat consumption in Australia [26], very few *Salmonella* isolates from sheep isolates were available, forcing us to combine sheep, cattle, and goats into a single category for source attribution purposes. However, the relatively low consumption of sheep meat and the low prevalence of *Salmonella* in sheep [25] suggest that sheep are unlikely to be a major source of *Salmonella* infections in NSW. We only had sufficient samples to estimate the relative frequency of serotypes over time in poultry which may have influenced the estimated trend in source attribution. Finally, we had only limited samples from wild, feral, and companion animals, which may have been especially important for the accurate attribution of rural cases. Though attribution to ‘unsampled’ sources includes these reservoirs, we are unable to estimate the contribution different species or groups of species.

## Conclusions

Layer chickens were the primary reservoir for human salmonellosis in NSW during 2009–2019; however, the importance of layers was less in rural populations and declined after 2015, concurrent with changes to food safety regulations and egg industry practices. The apparent increase in attribution to pigs and unsampled sources in 2017–2019 warrants further investigation. Our study highlights the need for a high-resolution typing method to be consistently applied to *Salmonella* isolates collected from humans, food, animals, and the environment. Using a consistent approach would allow for more precise estimates for source attribution analyses and assist in identifying specific sources in outbreak investigations. Moreover, the mobility of people and food products between Australian states and territories calls for a nationally unified approach to the surveillance of *Salmonella*. Furthermore, ongoing routine surveillance of *Salmonella* in food and food animals collected in a consistent sampling frame would improve the sample size and data quality for prevalence and source attribution estimates and allow these estimates to be updated regularly to monitor trends. Source attribution can inform and direct efforts to prevent foodborne disease. Source attribution analyses of data before and after interventions, public health responses, or changes to industry practices can help assess the effects of these changes. Regularly updated source attribution estimates would provide timely guidance for food safety authorities as novel *Salmonella* types or other epidemiological factors change the risk associated with specific animal reservoirs.

## Supplementary Information

**Additional file 1: Figure S1.** The observed distribution of common^1^ serotypes in human cases and the major food animal groups in NSW between 2008 and 2019. Each row shows the proportion of isolates due to each serotype in that source (number isolates from the source in parentheses). ^1^Serotypes within the twenty most common serotypes in one of the sources or in human cases. **Figure S2.** Source attribution proportions for a model ignoring differences over time, but adjusting for differences by rurality. (A) Attribution proportion for each of the major source groups for cases residing in different rurality zones. (B) The difference in attribution proportion by rurality with residents of major cities as the reference. Dots indicate posterior mean values, while dark and faint lines indicate 80% and 95% credible intervals, respectively. See Figure 2 in main text for model adjusting for rurality and changes over time. **Figure S3.** Attribution proportion for each of the major source groups for cases residing in different rurality zones over time. Dots and crosses indicate mean and median values, while dark and faint lines indicate 80% and 95% credible intervals, respectively. (Compare Figure 2A). **Figure S4.** The difference in attribution proportion by rurality with residents of major cities as the reference. Dots and crosses indicate mean and median values, while dark and faint lines indicate 80% and 95% credible intervals, respectively. (Compare Figure 2B). **Figure S5.** Posterior estimates of the relative efficiency (q) of included serotypes in a model including rurality, year-group, and an unsampled source (see Figures S4 and S5), with *S. infantis* used as a reference. High relative efficiency indicates a serotype more likely to lead to human disease (e.g. due to high virulence or high survivability) while low relative efficiency indicates serotypes that are common in source animals but rarely cause disease. Dots indicate median values, while thick and thin lines indicate 80% and 95% credible intervals, respectively. **Table S1.** The percentage of Salmonella isolates of serotype Typhimurium and the percentage of *S. typhimurium* isolates that were subtyped using phage-typing or MLVA typing. NA indicates that there were no *S. typhimurium* for the combination of source and year.

## Data Availability

The data used in this study were provided by New South Wales Department of Primary Industries, National Enteric Pathogens Surveillance Scheme, and Health Protection New South Wales; however, restrictions apply to the availability of these data, which were used under agreement for the current study, and so are not publicly available. Data are however available from the authors upon reasonable request and with permission of the relevant third parties.

## Abbreviations

NSW: New South Wales
CrI: Credible Interval
AUD: Australian Dollars
MLST: Multi-locus sequence typing
MLVA: Multiple-locus variable-number tandem repeat analysis
SA1: Australian Bureau of Statistics 2011 Statistical Area 1
SA2: Australian Bureau of Statistics 2011 Statistical Area 2
NEPSS: National Enteric Pathogens Surveillance System
NSWFA: New South Wales Food Authority
ESAM: *E. coli* And *Salmonella* Monitoring Program
ABARES: Australian Bureau of Agricultural and Resource Economics and Sciences
