# Supplementary material for: Source attribution of salmonellosis by time and geography in New South Wales, Australia
Source: BMC Infect Dis. 2022 Jan 4;22:14. doi: 10.1186/s12879-021-06950-7 (PMC8725445; doi:10.1186/s12879-021-06950-7)
Supplement: Supplementary file 1 — Additional file 1: Figure S1. The observed distribution of common1 serotypes in human cases and the major food animal groups in NSW between 2008 and 2019. Each row shows the proportion of isolates due to each serotype in that source (number isolates from the source in parentheses). 1Serotypes within the twenty most common serotypes in one of the sources or in human cases. Figure S2. Source attribution proportions for a model ignoring differences over time, but adjusting for differences by rurality. (A) Attribution proportion for each of the major source groups for cases residing in different rurality zones. (B) The difference in attribution proportion by rurality with residents of major cities as the reference. Dots indicate posterior mean values, while dark and faint lines indicate 80% and 95% credible intervals, respectively. See Figure 2 in main text for model adjusting for rurality and changes over time. Figure S3. Attribution proportion for each of the major source groups for cases residing in different rurality zones over time. Dots and crosses indicate mean and median values, while dark and faint lines indicate 80% and 95% credible intervals, respectively. (Compare Figure 2A). Figure S4. The difference in attribution proportion by rurality with residents of major cities as the reference. Dots and crosses indicate mean and median values, while dark and faint lines indicate 80% and 95% credible intervals, respectively. (Compare Figure 2B). Figure S5. Posterior estimates of the relative efficiency (q) of included serotypes in a model including rurality, year-group, and an unsampled source (see Figures S4 and S5), with S. infantis used as a reference. High relative efficiency indicates a serotype more likely to lead to human disease (e.g. due to high virulence or high survivability) while low relative efficiency indicates serotypes that are common in source animals but rarely cause disease. Dots indicate median values, while thick and thin lines indicate 80% and [file 12879_2021_6950_MOESM1_ESM.docx]

# Supplementary Materials


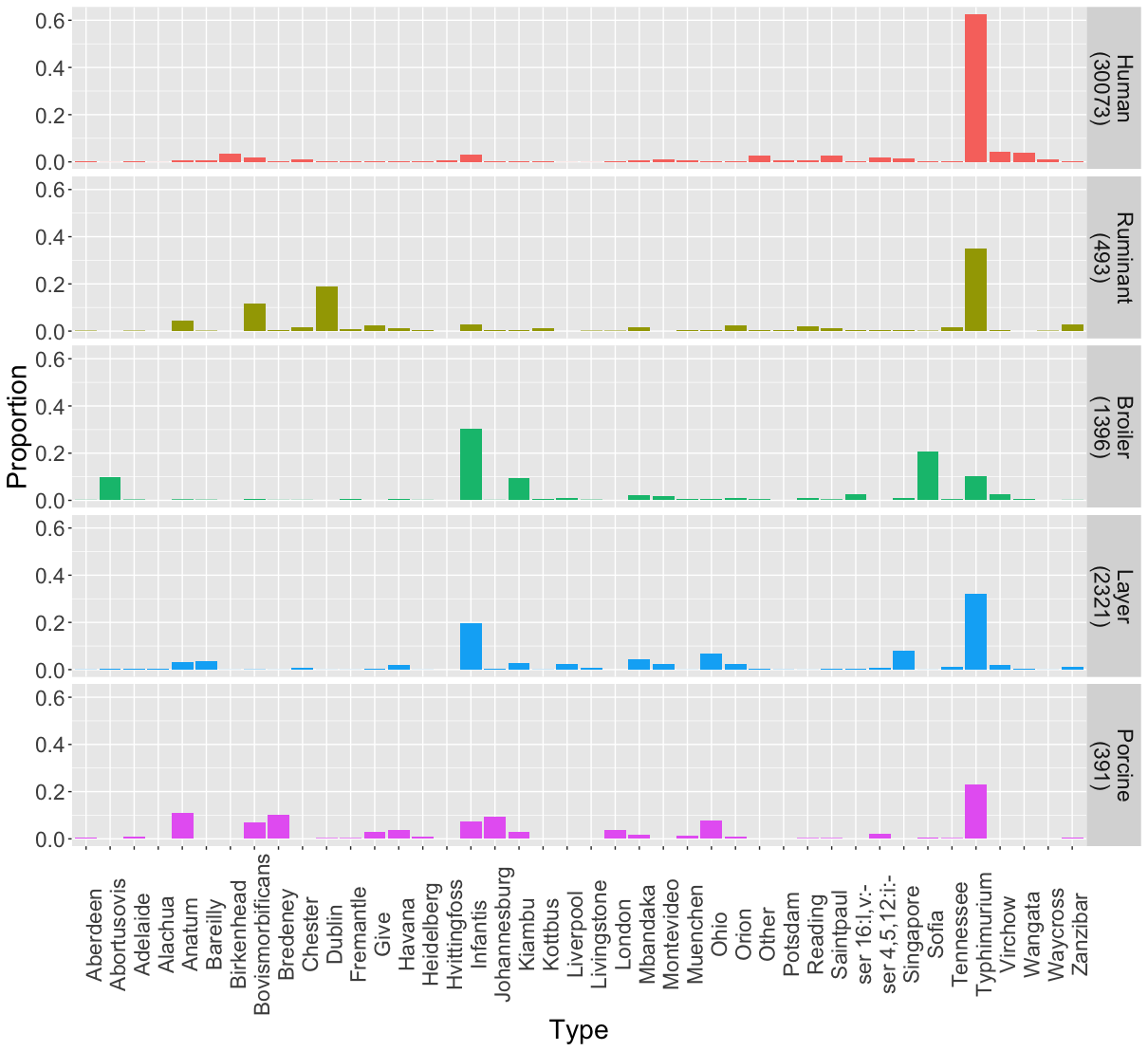


Serotype

**Figure S1**: The observed distribution of common^1^ serotypes in human cases and the major food animal groups in NSW between 2008 and 2019. Each row shows the proportion of isolates due to each serotype in that source (number isolates from the source in parentheses).

^1^ Serotypes within the twenty most common serotypes in one of the sources or in human cases.

**
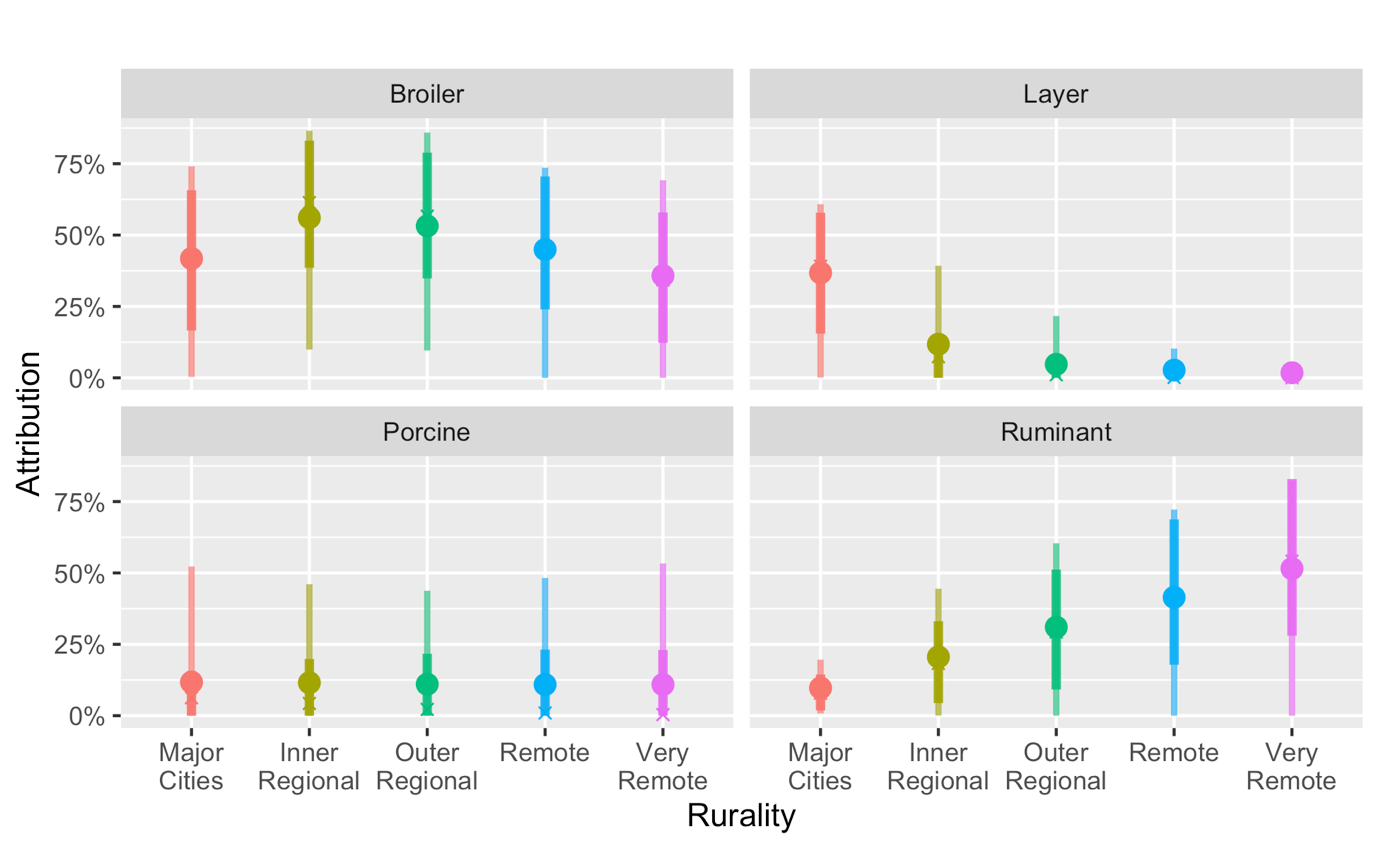

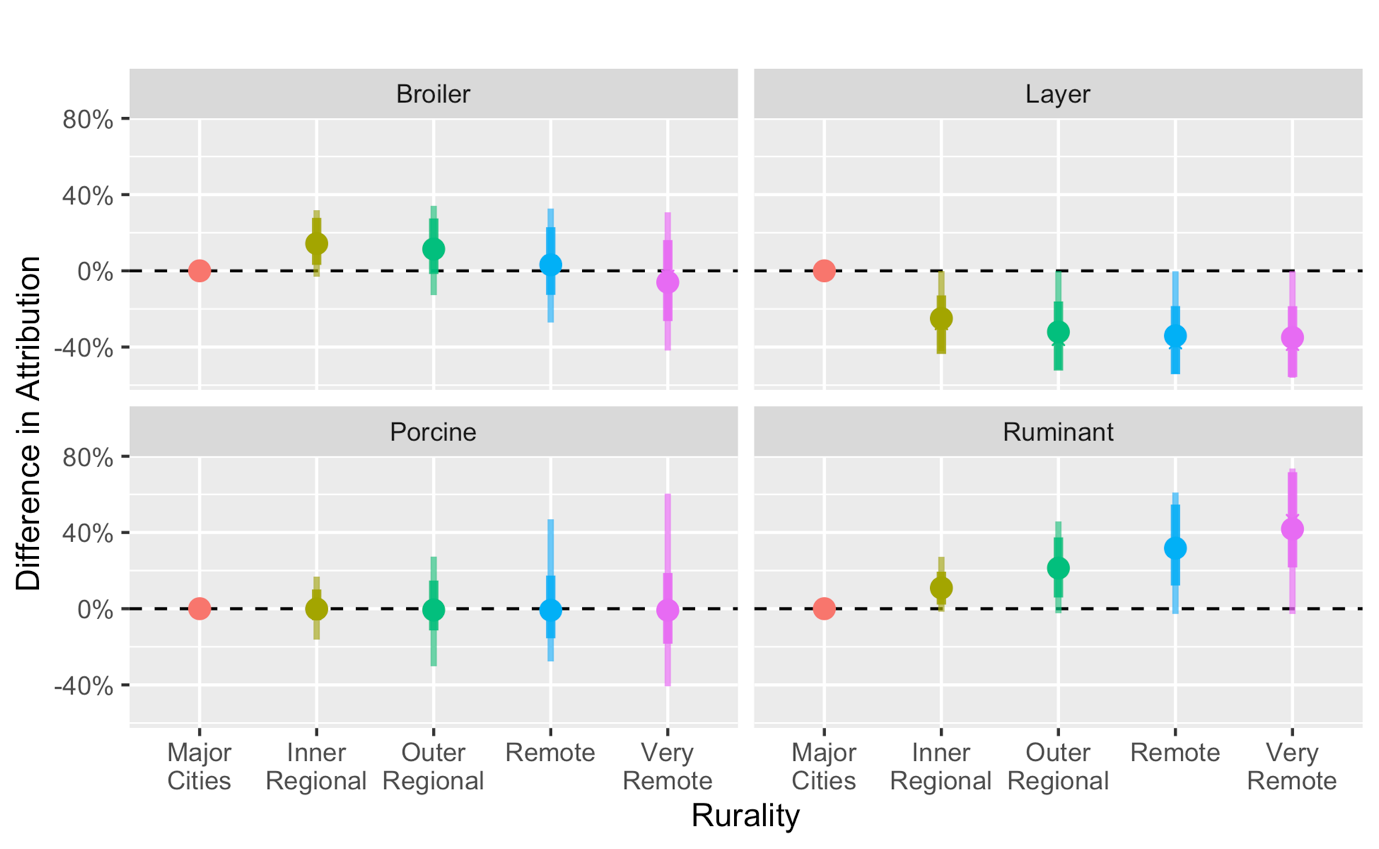
**

**A**

**B**

**Figure S2**: Source attribution proportions for a model ignoring differences over time, but adjusting for differences by rurality. (A) Attribution proportion for each of the major source groups for cases residing in different rurality zones. (B) The difference in attribution proportion by rurality with residents of major cities as the reference. Dots indicate posterior mean values, while dark and faint lines indicate 80% and 95% credible intervals, respectively. See Figure 2 in main text for model adjusting for rurality and changes over time.


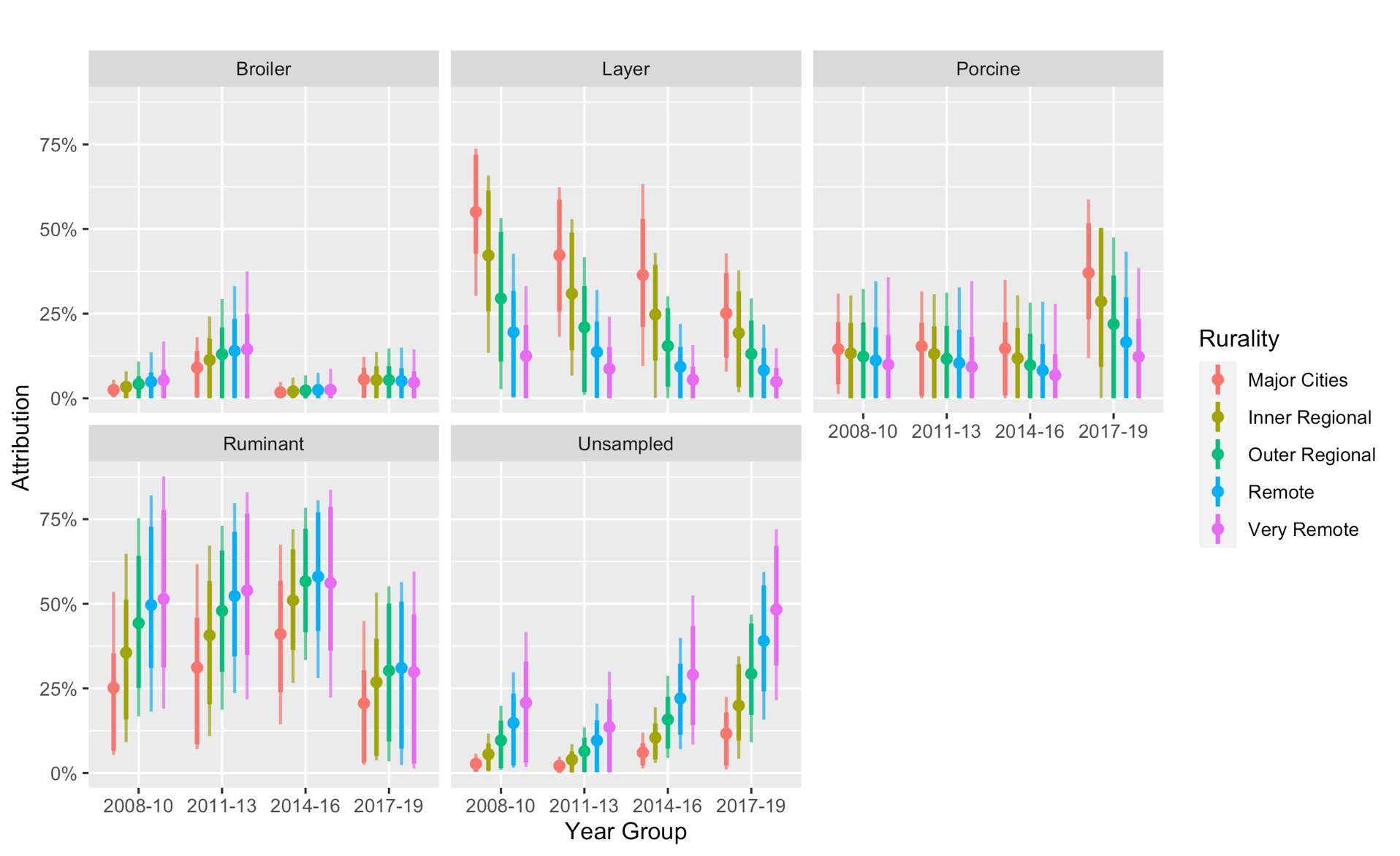


**Figure S3**: Attribution proportion for each of the major source groups for cases residing in different rurality zones over time. Dots and crosses indicate mean and median values, while dark and faint lines indicate 80% and 95% credible intervals, respectively. (Compare Figure 2A)


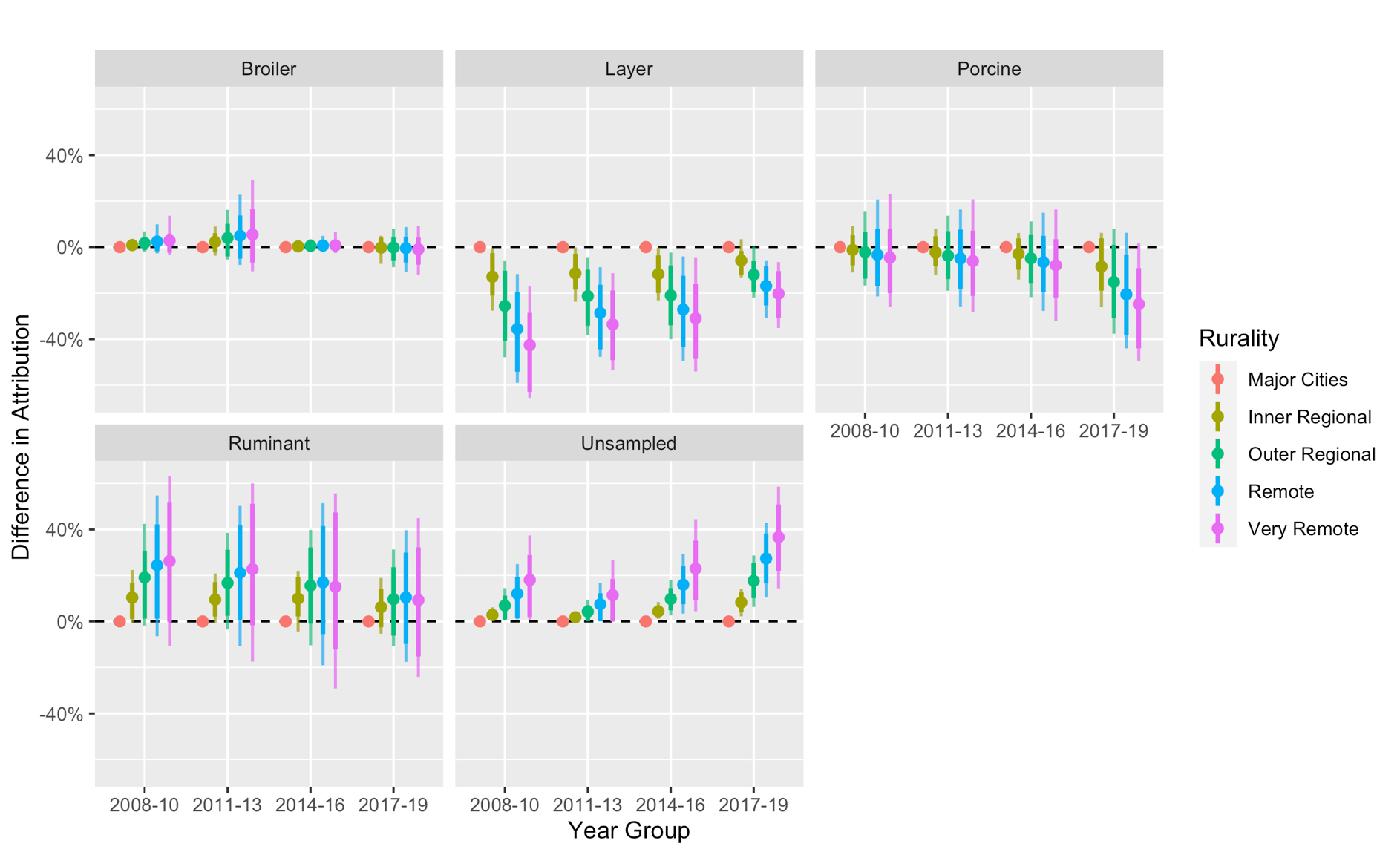


**Figure S4**: The difference in attribution proportion by rurality with residents of major cities as the reference. Dots and crosses indicate mean and median values, while dark and faint lines indicate 80% and 95% credible intervals, respectively. (Compare Figure 2B).


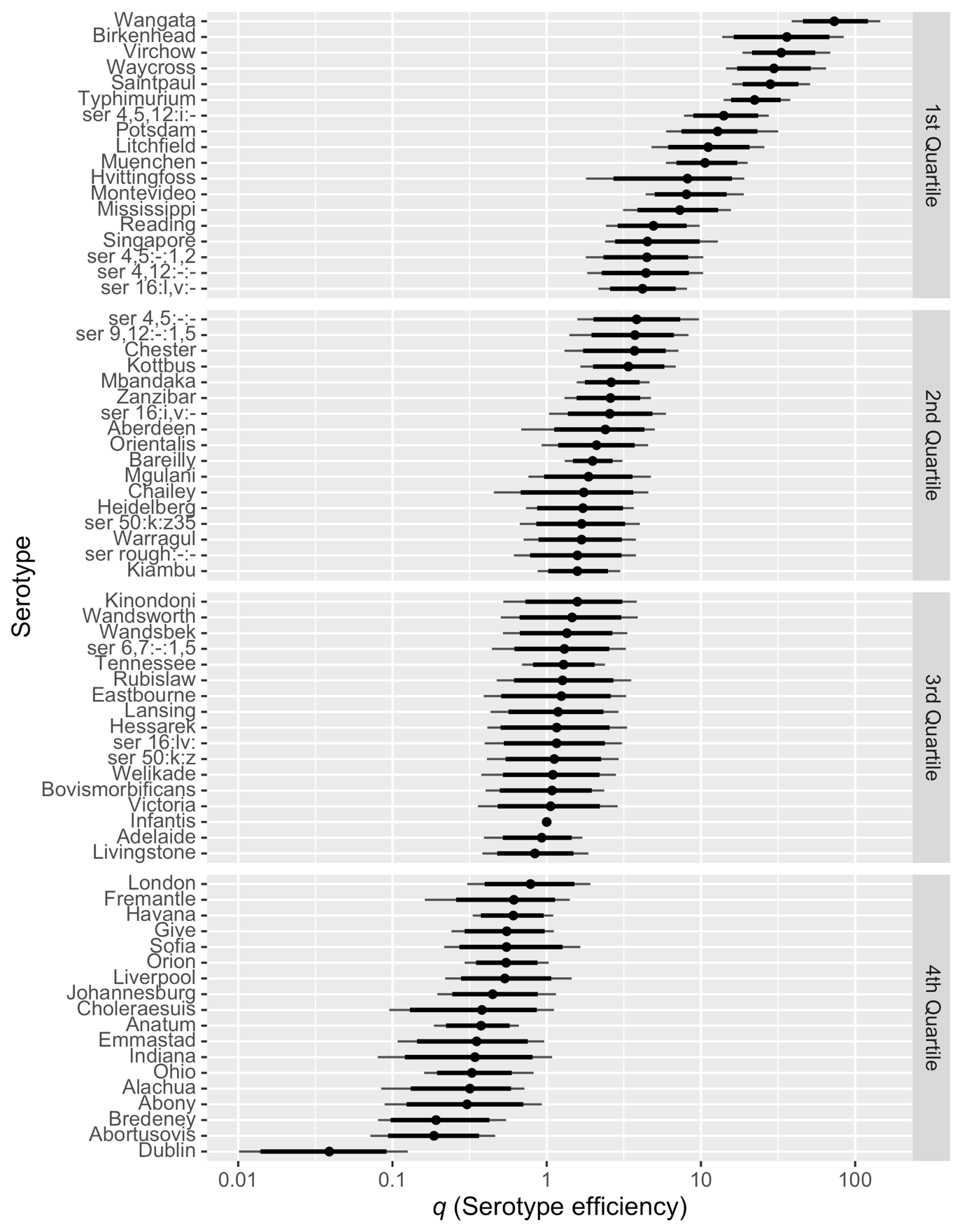


**Figure S5**: Posterior estimates of the relative efficiency ($q$) of included serotypes in a model including rurality, year-group and an unsampled source (see Figures S4 and S5), with *S.* Infantis used as a reference. High relative efficiency indicates a serotype more likely to lead to human disease (e.g. due to high virulence or high survivability) while low relative efficiency indicates serotypes that are common in source animals but rarely cause disease. Dots indicate median values, while thick and thin lines indicate 80% and 95% credible intervals, respectively

**Table S1:** The percentage of *Salmonella* isolates of serotype Typhimurium and the percentage of *S.* Typhimurium isolates that were subtyped using phage-typing or MLVA typing. NA indicates that there were no *S.* Typhimurium for the combination of source and year.

|  | Year | | | | | | | | | | | |  |
| --- | --- | --- | --- | --- | --- | --- | --- | --- | --- | --- | --- | --- | --- |
|  | **2008** | **2009** | **2010** | **2011** | **2012** | **2013** | **2014** | **2015** | **2016** | **2017** | **2018** | **2019** | **All** |
| % Typhimurium |  |  |  |  |  |  |  |  |  |  |  |  |  |
| Human cases | 65.0 | 71.1 | 71.9 | 71.1 | 69.6 | 67.3 | 73.9 | 62.1 | 51.4 | 46.4 | 42.9 | 44.8 | 62.5 |
| Ruminants | 22.7 | 32.8 | 31.0 | 39.4 | 32.8 | 40.0 | 35.7 | 37.0 | 72.7 | 50.0 | 100.0 | 0.0 | 34.9 |
| Broilers | 0.7 | 9.1 | 16.3 | 19.7 | 32.5 | 10.8 | 8.2 | 3.6 | 3.2 | 13.1 | 14.3 | 21.1 | 10.1 |
| Layers | 39.6 | 44.4 | 42.6 | 31.5 | 33.8 | 38.2 | 33.8 | 34.8 | 22.9 | 18.4 | 3.1 | 24.5 | 32.1 |
| Pigs | 25.0 | 61.5 | 33.3 | 59.6 | 11.1 | 15.2 | 8.7 | 12.5 | 19.6 | 0.0 | 0.0 | 0.0 | 23.0 |
| % Typhimurium Phage typed |  |  |  |  |  |  |  |  |  |  |  |  |  |
| Human cases | 97.6 | 97.7 | 83.2 | 3.6 | 6.0 | 3.7 | 1.4 | 3.3 | 1.3 | 0.4 | 0.1 | 0.4 | 25.1 |
| Ruminants | 100.0 | 100.0 | 50.0 | 92.7 | 95.5 | 64.3 | 50.0 | 17.6 | 25.0 | 66.7 | 100.0 | NA | 74.4 |
| Broilers | 100.0 | 100.0 | 87.5 | 80.0 | 78.9 | 100.0 | 100.0 | 7.7 | 0.0 | 54.5 | 0.0 | 0.0 | 58.2 |
| Layers | 100.0 | 100.0 | 87.9 | 64.7 | 44.9 | 61.5 | 93.4 | 92.7 | 84.0 | 32.0 | 75.0 | 5.2 | 73.3 |
| Pigs | 100.0 | 100.0 | 71.4 | 85.7 | 100.0 | 100.0 | 0.0 | 100.0 | 60.0 | NA | NA | NA | 84.4 |
| % Typhimurium MLVA typed |  |  |  |  |  |  |  |  |  |  |  |  |  |
| Human cases | 91.1 | 93.4 | 91.5 | 94.3 | 92.8 | 91.2 | 95.3 | 89.9 | 94.4 | 93.3 | 98.2 | 86.0 | 92.9 |
| Ruminants | 26.7 | 9.1 | 0.0 | 9.8 | 18.2 | 28.6 | 40.0 | 11.8 | 25.0 | 66.7 | 50.0 | NA | 16.9 |
| Broilers | 0.0 | 0.0 | 0.0 | 0.0 | 0.0 | 0.0 | 20.0 | 7.7 | 0.0 | 90.9 | 0.0 | 0.0 | 8.5 |
| Layers | 14.9 | 0.0 | 0.0 | 0.0 | 10.1 | 60.4 | 89.5 | 90.9 | 84.0 | 28.0 | 50.0 | 10.3 | 31.7 |
| Pigs | 20.0 | 0.0 | 0.0 | 28.6 | 40.0 | 75.0 | 0.0 | 100.0 | 30.0 | NA | NA | NA | 32.2 |
